# Identification and validation of plant height, spike length and spike compactness loci in common wheat (*Triticum aestivum* L.)

**DOI:** 10.1186/s12870-022-03968-0

**Published:** 2022-12-06

**Authors:** Hong Liu, Zhipeng Shi, Feifei Ma, Yunfeng Xu, Guohao Han, Jinpeng Zhang, Dongcheng Liu, Diaoguo An

**Affiliations:** 1 Center for Agricultural Resources Research, Institute of Genetics and Developmental Biology, Chinese Academy of Sciences, Shijiazhuang, Hebei 050022 China; 2 State Key Laboratory of North China Crop Improvement and Regulation, College of Agronomy, Hebei Agricultural University, Baoding, 071000 Hebei China; 3 The National Key Facility for Crop Gene Resources and Genetic Improvement, Institute of Crop Science, Chinese Academy of Agricultural Sciences, Beijing, 100081 China; 4 The Innovative Academy for Seed Design, Chinese Academy of Sciences, Beijing, 100101 China; 5 University of Chinese Academy of Sciences, Beijing, 100049 China

**Keywords:** Plant height, Spike length, Spike compactness, Quantitative trait locus, *Triticum aestivum* L

## Abstract

**Background:**

Plant height (PH), spike length (SL) and spike compactness (SCN) are important agronomic traits in wheat due to their strong correlations with lodging and yield. Thus, dissection of their genetic basis is essential for the improvement of plant architecture and yield potential in wheat breeding. The objective of this study was to map quantitative trait loci (QTL) for PH, SL and SCN in a recombinant inbred line (RIL) population derived from the cross ‘PuBing3228 × Gao8901’ (PG-RIL) and to evaluate the potential values of these QTL to improve yield.

**Results:**

In the current study, Five, six and ten stable QTL for PH, SL, and SCN, respectively, were identified in at least two individual environments. Five major QTL *QPh.cas-5A.3*, *QPh.cas-6A*, *QSl.cas-6B.2*, *QScn.cas-2B.2* and *QScn.cas-6B* explained 5.58–25.68% of the phenotypic variation. Notably, two, three and three novel stable QTL for PH, SL and SCN were identified in this study, which could provide further insights into the genetic factors that shape PH and spike morphology in wheat. Conditional QTL analysis revealed that QTL for SCN were mainly affected by SL. Moreover, a Kompetitive Allele Specific PCR (KASP) marker tightly linked to stable major QTL *QPh.cas-5A.3* was developed and verified using the PG-RIL population and a natural population.

**Conclusions:**

Twenty-one stable QTL related to PH, SL, and SCN were identified. These stable QTL and the user-friendly marker *KASP8750* will facilitate future studies involving positional cloning and marker-assisted selection in breeding.

**Supplementary Information:**

The online version contains supplementary material available at 10.1186/s12870-022-03968-0.

## Background

Common wheat (*Triticum aestivum* L.) is one of the most important crop worldwide and provides approximately 20% of the calories in the humans diet [1]. As the world population is growing continuously, increasing wheat production is an ongoing major goal for wheat breeding [2]. Wheat yield is determined by the number of spikes, kernel number per spike (KNS) and thousand kernel weight (TKW) [3]. Also, plant height (PH), spike length (SL) and spike compactness (SCN) are closely related to KNS and TKW [4, 5]. Thus, PH, SL, and SCN are important selection indicators used in high-yield breeding [6].

PH is closely associated with lodging resistance and grain yield in wheat [7]. The application of green revolution genes (*Rht-B1b* and *Rht-D1b*) has result in several new cultivars that, were not prone to lodging under increased fertilizer application, thereby successfully achieving increased yield [8]. However, the green revolution genes *Rht-B1b* and *Rht-D1b* also decreased KNS and TKW while reduce PH [9]. To date, the number of major genes which affect PH in wheat and without causing substantial deleterious agronomic effects, is not large [10]. Therefore, the exploration and utilization of new dwarfing QTL/genes have been a major focus in wheat research.

QTL mapping is an efficient strategy for detecting QTL and genes for PH [11]. Twenty-five *Rht* genes distributed on 11 wheat chromosomes have been identified and formally named [12]. *Rht-B1b*, *Rht-D1b*, *Rht8*, *Rht13* and *Rht24* were widely used in modern cultivars [7, 13–14]. Several *Rht* genes regulating PH have been cloned in wheat. Among them, *Rht12* encodes a gibberellin (GA) 2-β-dioxygenase [15], *Rht23* likely encodes an AP2 transcription factor [16], *Rht24b* encodes a GA 2-oxidase [17], and *Rht8* encodes a ribonuclease H-like protein [18, 19]. Additionally, several other genes regulating PH have been cloned using comparative genomics and genome wide association study approaches, including *TaDEP1* [20], *TaCOLD1* [21], *TaTB1* [10, 22], and *TaARF12* [23].

SL and SCN are important spike morphology traits closely related to KNS and TKW in wheat [5]. To date, only a few genes that regulate SL and SCN have been cloned in wheat. For instance, *Q* encodes AP2 domain transcription factor, which interact with miRNA172 to regulate brittle spike, SL, SCN, and grain shattering [2, 24]. *Rht24b*, *Rht8*, and *TaARF12* have multiple functions and could regulate PH and SL [17, 18, 23]. Many QTL related to SL and SCN have been reported using linkage analysis and association analysis. The major stable QTL for SL and SCN were mainly distributed on wheat chromosomes 2D, 3A, 4A, 4B, 5A, 6A, 6B, 7A, 7B and 7D [2, 25, 26, 27, 28, 29]. *QSpl.nau-2D*, a major QTL for SL on chromosome 2D, was found to affect SL, SCN, and TKW [4]. Low SCN can reduce the severity of fusarium head blight (FHB), which is a major disease that significantly impacts wheat production [30, 31]. Since SL and SCN are closely related to important traits such as yield and FHB, markers tightly linked to these regions can be used in marker-assisted selection breeding and positional cloning. However, although many QTL for SL and SCN have been reported, the important QTL available for wheat breeding are still limited.

The wheat germplasm PuBing 3228 (P3228), which has superior features such as large spikes, was widely used in the main growing areas of winter wheat of China. Gao 8901 (G8901) is a commercial cultivar in Yellow and Huai River valley winter wheat region of China with a shorter PH and medium size spike when comparing with P3228. Here, we aimed to (i) identify QTL for PH, SL, and SCN using a RIL population derived from ‘P3228 × G8901’ (PG-RIL); (ii) reveal the effect of SL to PH and to SCN, respectively, using conditional QTL analysis; (iii) detect QTL clusters or pleiotropic loci associated with those traits and (iv) develop a Kompetitive Allele Specific PCR (KASP) marker for stable QTL to be used in marker-assisted selection (MAS) in wheat breeding.

## Results

### Phenotypic performance and correlation analysis

**Fig. 1:**
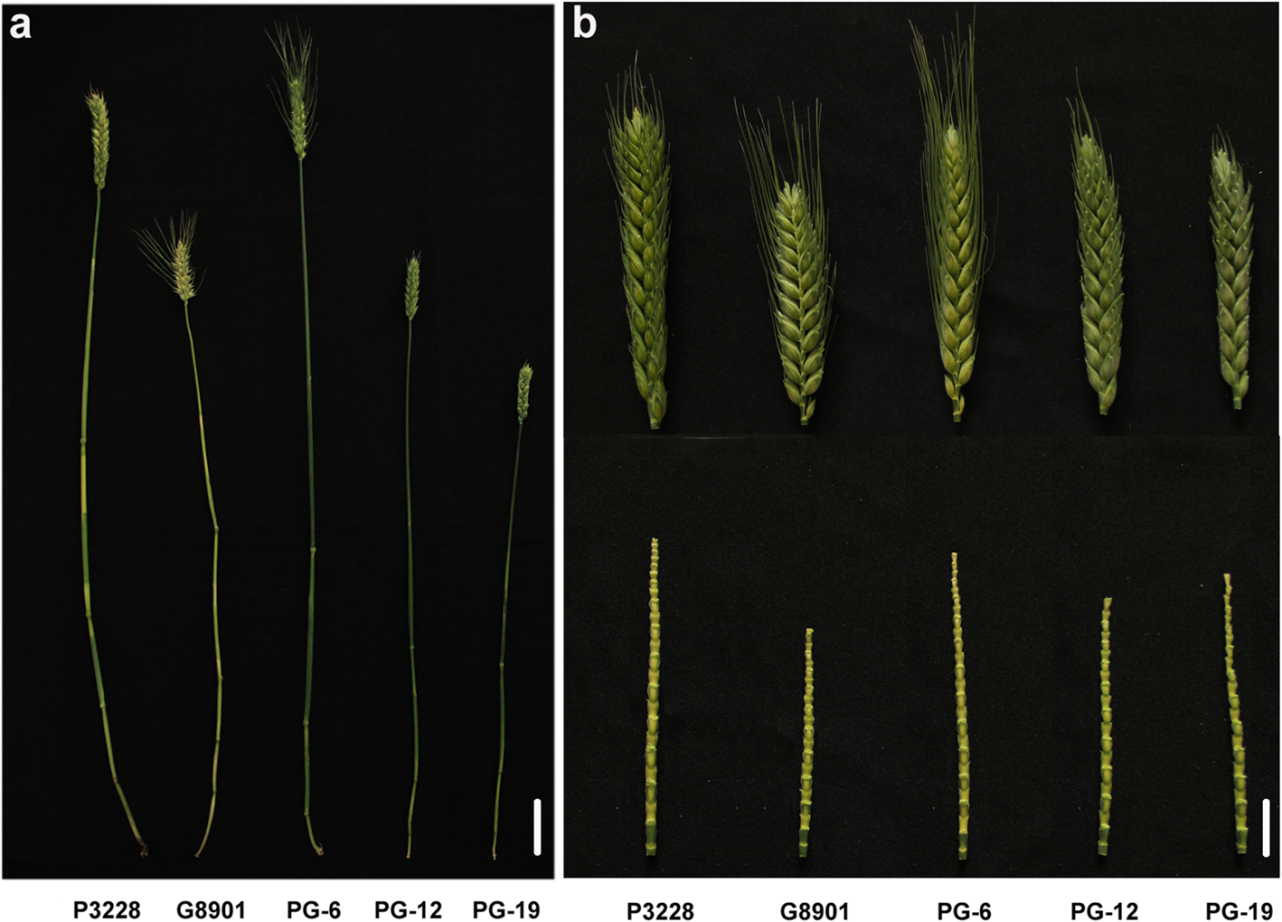
Plant height **a**, spike length and spike compactness **b** of two parents P3228 and G8901, and some representative RIL

**Fig. 2:**
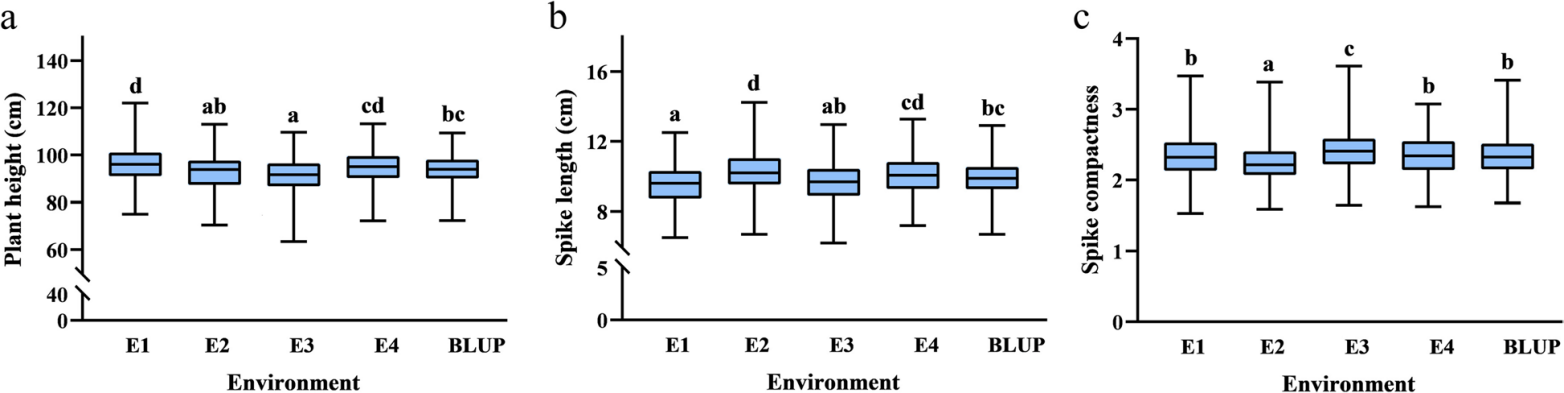
Frequency distribution of plant height **a**, spike length **b** and spike compactness **c** in RIL population of P3228 and G8901 in BLUP datasets

**Fig. 3:**
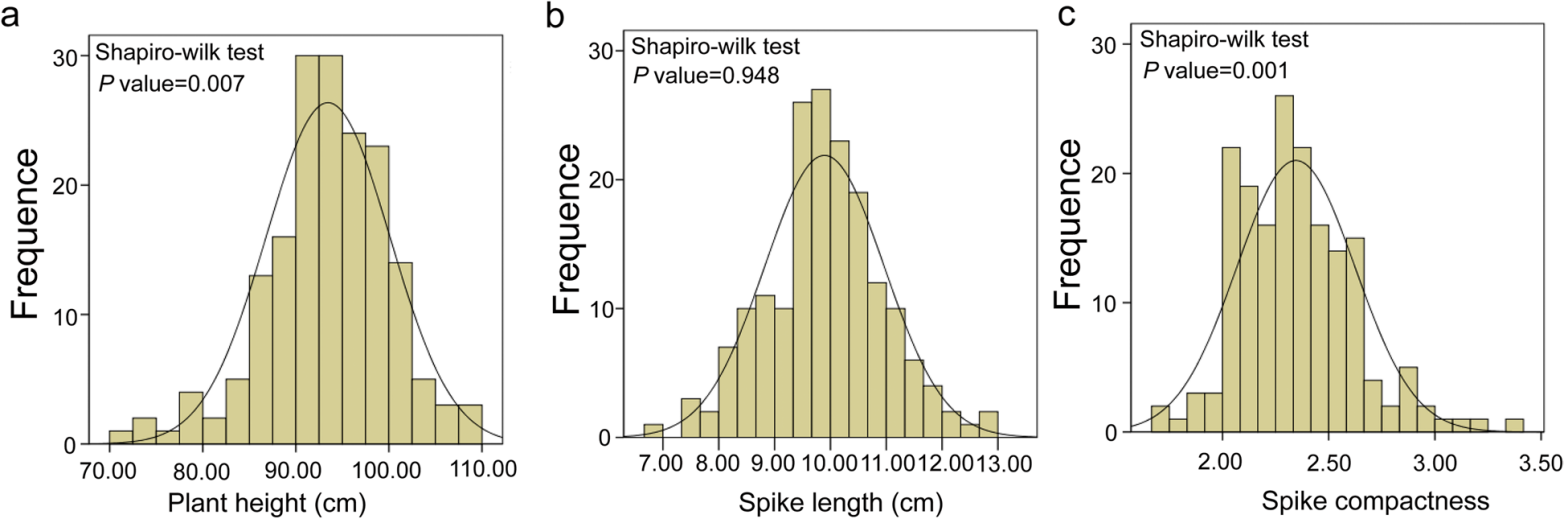
Phenotype of plant height **a**, spike length **b** and spike compactness **c** in four environments and BLUP datasets. Multiple comparative analyses of the three traits (LSD) were carried out in different environments

The 176 RIL population and the two parents were planted in four environments. The two parents P3228 and G8901 had significant differences in PH, SL, and SCN. Compared with G8901, P3228 had a taller PH and longer SL but a lower SCN (Fig. 1 and Table 1). Transgressive segregation was common at both ends of the distribution for PH, SL, and SCN (Table 1 and Fig. 2a-c). The variance showed highly significant effects of genotype, environment, and genotype × environment (G × E) interaction for PH, SL, and SCN (Additional file 1: Table S1). Genotype RIL046, RIL145, and RIL149 gave significantly highest PH, SL, and SCN in comparison to all other genotypes, respectively (Additional file 1: Table S2-S4). Likewise, PH, SL, and SCN was significantly higher in environment E1, E2, and E2 as compared to other environments, respectively (Fig. 3a-c). Moreover, their interactions were also significant where marked increased was recorded for genotype RIL046 for PH in environment E1, RIL145 for SL in environment E2, and RIL149 for SCN in environment E2, respectively (Fig. 3 and Additional file 1: Table S1-S4). The PH, SL, and SCN showed high broad-sense heritability at 0.78, 0.87, and 0.89, respectively. (Table 1). The best linear unbiased predictors (BLUP) datasets for each trait showed a normal distribution based on the Shapiro–Wilk test and Pearson’s correlation coefficients, suggesting the polygenic inheritance of these traits (Table 2).

**Table 1:** Phenotypes of the parents and PG-RIL population in this study

Trait	Environment	Parents		PG-RILs					
		P3228	G8901	Minimum	Maximum	Mean	SD	CV(%)	*H*^*2*^
PH	E1	101.00	89.67	75.00	122.00	95.74	8.33	8.70	0.78
	E2	98.00	80.67	70.33	113.00	92.50	7.96	8.61	
	E3	92.10	78.60	63.40	109.60	91.11	7.63	8.37	
	E4	96.70	85.70	72.20	113.20	94.46	7.99	8.46	
	BLUP	96.95	83.66	72.30	109.30	93.46	6.64	7.10	
SL	E1	10.40	7.77	6.50	12.50	9.60	1.15	11.98	0.87
	E2	11.47	7.98	6.70	14.23	10.24	1.18	11.52	
	E3	10.10	8.12	6.20	12.96	9.71	1.20	12.36	
	E4	10.34	9.01	6.06	13.28	10.00	1.90	19.00	
	BLUP	10.58	8.22	6.69	12.91	9.90	1.07	10.81	
SCN	E1	2.21	2.78	1.53	3.47	2.34	0.32	13.68	0.89
	E2	2.01	2.75	1.59	3.39	2.25	0.28	12.44	
	E3	2.34	2.67	1.65	3.61	2.43	0.31	12.76	
	E4	2.23	2.52	1.63	3.63	2.38	0.31	13.03	
	BLUP	2.20	2.68	1.68	3.41	2.35	0.28	11.91	

*PH *Plant height, *SL *Spike length, *SCN *Spike compactness

**Table 2:** Correlation coefficients among the plant height, spike length and spike compactness of PG-RIL population in four environments and BLUP datasets

Trait	Blup		EL		E2		E3		E4	
	PH	SL	PH	SL	PH	SL	PH	SL	PH	SL
SL	0.299^**^		0.201^**^		0.294^**^		0.298^**^		0.306^**^	
SCN	-0.349	-0.885	-0.213	-0.805	-0.303	-0.864	-0.369	-0.901	-0.335	-0.885

*BLUP *Best linear unbiased predictors, *PH *Plant height, *SL *Spike length, *SCN *Spike compactness. ^*^significant at *P* < 0.05 level; ^**^significant at *P* < 0.01 level

### QTL mapping

**Fig. 4:**
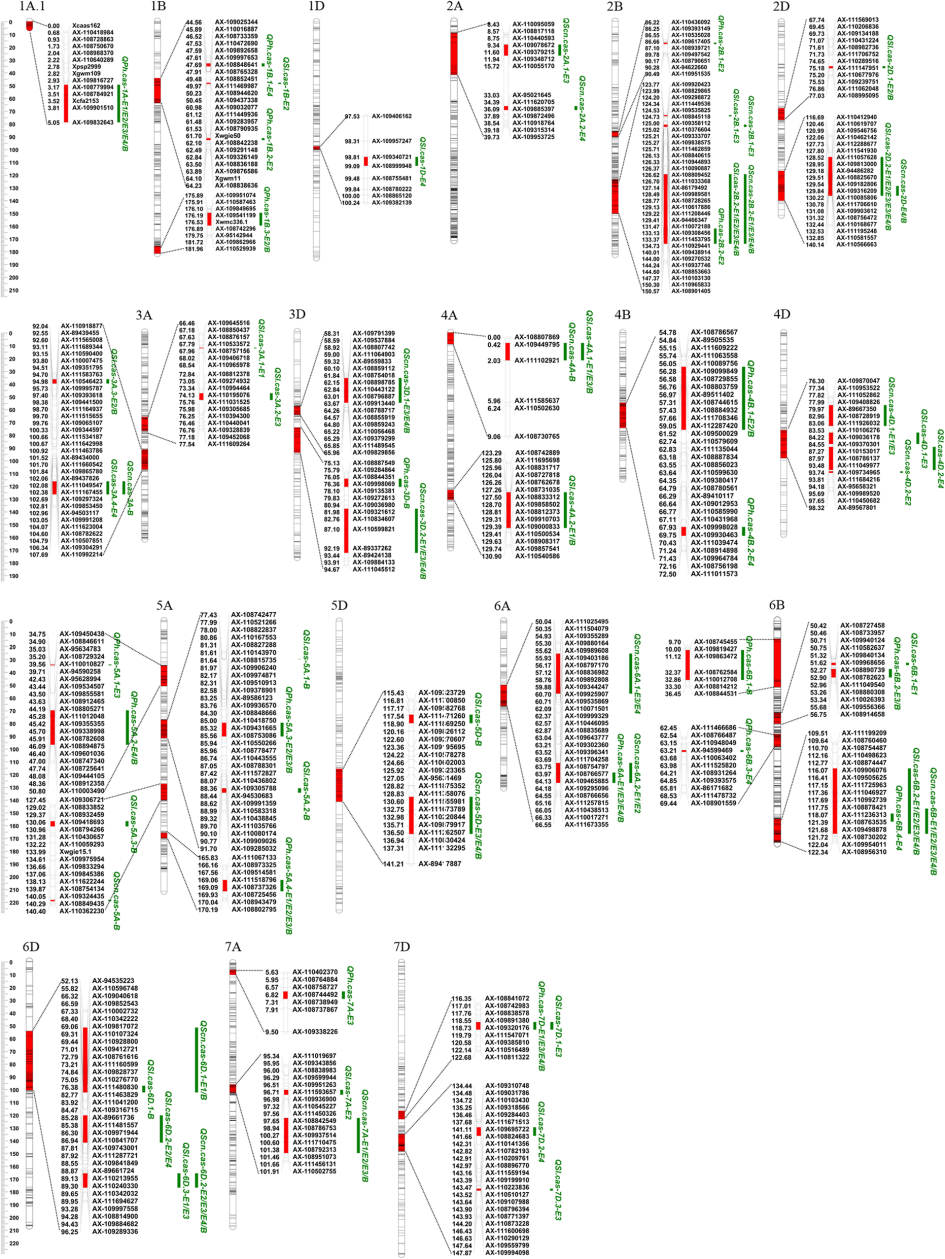
Genetic locations of QTL intervals associated with plant height, spike length and spike compactness. Uniform centimorgan (cM) scales are shown on the left. QTL are indicated on the right side of each chromosome. For QTL detected in different environments, a slash is inserted to distinguish the environments. The codes E1, E2, E3, E4 and B represent QTL detected in 2013LC, 2014LC, 2015LC, 2016LC environments and BLUP datasets, respectively

A total of 68 putative QTL were detected for PH, SL, and SCN (Fig. 3 and Additional file 1: Table S2). Among them, 27, 19, and 22 QTL were located on the A, B, and D subgenomes, respectively. The single QTL explained 1.05–30.19% of the phenotypic variance with threshold log-of-odds (LOD) values ranging from 2.74 to 27.28 (Additional file 1: Table S2). Twenty-one stable QTL could be detected in at least two individual environments (Fig. 4 and Table 3).

**Table 3:** Stable QTL for plant height, spike length and spike compactness in the PG-RIL population

Trait	QTL	Env	Markers Interval	Genetic Interval (cM)	PVE%	Add	References
PH	*QPh.cas-1A.1*	E1	*AX-109816727* –*AX-109832643*	2.932–5.047	6.730	-3.482	
		E2			3.732	-2.609	
		E3			10.230	-3.632	
		E4			8.697	-3.947	
		BLUP			7.981	-2.945	
	*QPh.cas-5A.3*	E2	*AX-109936570* *–AX-110418750*	83.758–85.001	17.901	3.652	
		E3			9.665	2.295	
		BLUP			8.381	1.934	
	*QPh.cas-5A.4*	E1	*AX-109514581* –*AX-111518796*	167.560–169.057	4.307	1.851	
		E2			3.656	1.629	
		E3			3.597	1.381	
		BLUP			4.596	1.413	
	*QPh.cas-6A*	E1	*AX-108766577* –*AX-111257815*	63.966–65.160	9.977	2.814	
		E3			11.401	2.463	
		E4			17.037	3.439	
		BLUP			13.248	2.397	
	*QPh.cas-7D*	E1	*AX-109320176* *–AX-111547071*	118.725–119.787	7.262	2.402	[5]
		E3			9.459	2.238	
		E4			9.721	2.600	
		BLUP			8.421	1.912	
SL	*QSl.cas-2B.2*	E1	*AX-110929441* *–AX-110103130*	134.726–147.365	7.054	0.394	[32]
		E2			3.590	0.213	
		E3			2.989	0.273	
		E4			7.250	0.323	
		BLUP			4.134	0.222	
	*QSl.cas-2D.2*	E1	*AX-110462142* –*AX-110168677*	122.055–132.439	3.786	-0.292	[33]
		E2			7.479	-0.310	
		E3			27.288	-0.830	
		E4			5.516	-0.284	
		BLUP			10.051	-0.351	
	*QSl.cas-4A.1*	E1	*AX-109449795* –*AX-111102921*	0.421–2.028	3.603	-0.282	
		E3			1.237	-0.176	
		BLUP			5.141	-0.248	
	*QSl.cas-6B.2*	E1	*AX-108874447* –*AX-108763535*	112.768–121.387	5.577	0.351	[6, 34]
		E2			22.939	0.543	
		E3			13.152	0.576	
		E4			25.676	0.613	
		BLUP			18.651	0.476	
	*QSl.cas-6D.2*	E2	*AX-111480830* –*AX-111463829*	76.375–82.772	5.455	-0.270	
		E4			5.015	-0.276	
	*QSl.cas-6D.3*	E1	*AX-111694627* –*AX-109997558*	89.948–93.275	3.488	-0.288	
		E3			4.364	-0.343	
SCN	*QScn.cas-2B.2*	E1	*AX-110929441* –*AX-110103130*	134.726–147.365	11.396	-0.102	
		E3			5.893	-0.068	
		E4			6.972	-0.076	
		BLUP			3.913	-0.050	
	*QScn.cas-3D.1*	E3	*AX-111064903* –*AX-108788717*	58.995–64.259	5.474	0.066	
		E4			4.505	0.061	
		BLUP			5.7222	0.061	
	*QScn.cas-3D.2*	E1	*AX-110834607* –*AX-89337262*	82.759–92.192	9.8091	0.094	[35]
		E3			4.3772	0.059	
		E4			5.2069	0.066	
		BLUP			4.0048	0.051	
	*QScn.cas-4D.1*	E1	*AX-109408826* –*AX-108728919*	77.985–82.964	5.080	0.068	[32, 36]
		E3			3.605	0.053	
	*QScn.cas-5D*	E3	*AX-111555981* –*AX-111262507*	130.600–136.499	2.6319	0.046	[32]
		E4			5.7563	0.069	
		BLUP			5.0313	0.057	
	*QScn.cas-6A.1*	E3	*AX-111504079* –*AX-109355289*	50.353–54.925	4.656	-0.062	[32]
		E4			3.5724	-0.056	
	*QScn.cas-6A.2*	E1	*AX-108835689* –*AX-111257815*	62.873–65.160	6.1232	-0.075	
		E2			6.9682	-0.072	
	*QScn.cas-6B*	E1	*AX-111236313* –*AX-108763535*	118.069–121.387	11.427	-0.101	
		E2			20.0194	-0.123	
		E3			21.9433	-0.133	
		E4			20.8836	-0.134	
		BLUP			24.213	-0.126	
	*QScn.cas-6D.2*	E2	*AX-111694627* –*AX-109997558*	89.948–93.275	8.227	0.081	[37]
		E3			9.2452	0.089	
		E4			11.5314	0.103	
		BLUP			6.1995	0.066	
	*QScn.cas-7A*	E1	*AX-108786753* –*AX-108792313*	98.942–101.378	6.5443	-0.077	
		E2			7.4825	-0.075	
		E3			3.4924	-0.053	
		BLUP			3.8594	-0.050	

*Env *Environments, *BLUP *Best linear unbiased predictors, *PVE *Phenotypic variance explained, *Add *Additive effect

A total of 21 QTL for PH were detected, of which 14 QTL carried alleles from G8901 that can increase PH, while the remaining seven alleles were from P3228 (Fig. 4 and Additional file 1: Table S2). In addition, five stable QTL were detected in at least two environments, including *QPh.cas-1A.1*, *QPh.cas-5A.3*, *QPh.cas-5A.4*, *QPh.cas-6A* and *QPh.cas-7D* (Table 3). Remarkably, *QPh.cas-1A.1* was detected in all the environments and BLUP datasets and explained 3.73% to 10.23% of the phenotypic variation, which represents this QTL may be less affected by the environment (Table 3). *QPh.cas-5A.3* was detected on the long arm of chromosome 5A in three environments and BLUP datasets, explaining 8.38% to 17.90% of the phenotypic variation (Table 3). *QPh.cas-5A.4* was also detected on chromosome 5AL in three environments and BLUP datasets, explaining 3.60% to 4.60% of the phenotypic variation (Table 3). The largest effect QTL was *QPh.cas-6A* located on the long arm of chromosome 6A. This QTL was detected in the three environments as well as the BLUP datasets, and the phenotypic variance explained (PVE) ranged from 9.98% to 17.04% (Fig. 4 and Table 3). Among these QTL, increased PH was contributed by the G8901 alleles for *QPh.cas-1A.1* and by the P3228 allele for *QPh.cas-5A.3*, *QPh.cas-5A.4*, *QPh.cas-6A* and *QPh.cas-7D*. The PVE value of stable QTL for PH indicated that the contribution of P3228 was greater than G8901.

Twenty-eight QTL for SL were detected, of which six QTL were significant in at least two environments (Table 3). Among the six stable QTL, the high SL allele of *QSl.cas-2B.2* and *QSl.cas-6B.2* was contributed by P3228, while the high SL allele of *QSl.cas-2D.2*, *QSl.cas-4A.1*, *QSl.cas-6D.2* and *QSl.cas-6D.3* was contributed by G8901. The stable major QTL *QSl.cas-6B.2* was detected on the long arm of chromosome 6B in all the environments and BLUP datasets and explained 5.58% to 25.68% of the phenotypic variation (Fig. 4 and Table 3). *QSl.cas-2B.2* and *QSl.cas-2D.2* were also detected in all the environments and BLUP datasets, with PVEs of 2.90–7.25% and 3.79–27.29%, respectively (Table 3).

For SCN, a total of 19 QTL were identified, with the PVE of individual QTL ranging from 2.42% to 24.22% (Fig. 3, Table 3 and Additional file 1: Table S2). Nine stable QTL were found in at least two environments. Among these stable QTL, increased SCN was contributed by *QScn.cas-2B.2*, *QScn.cas-6A.1, QScn.cas-6A.2, QScn.cas-6B* and *QScn.cas-7A* from G8901, and *QScn.cas-3D.1*, *QScn.cas-3D.2*, *QScn.cas-4D.1* and *QScn.cas-5D* from P3228 (Fig. 4 and Table 3). The stable major QTL *QScn.cas-6B* on the long arm of chromosome 6B was detected in four environments and BLUP datasets, explaining 10.98–24.21% of the phenotypic variance (Fig. 4 and Table 3). Notably, based on the QTL interval and peak marker positions, the QTL *QScn.cas-6B*, *QScn.cas-2B.2*, and *QScn.cas-6D.2* were mapped to the flanking regions of the QTL identified for SL, and *QScn.cas-6A.2* was colocalized with QTL *QPh.cas-6A* for PH (Fig. 4 and Table 3). These results suggested that these four regions contain either a single QTL with pleiotropic effects or more than one tightly linked QTL affecting pleiotropic effects.

### Conditional QTL analysis

To dissect the genetic effects of PH on the expression of QTL for SL, conditional QTL analysis was conducted. Thirteen conditional QTL comprising 25 QTL × environments in total affecting PH were detected for PH|SL (Table 4). Among them, 11 QTL were detected by unconditional QTL mapping, and two novel QTL, *QPh.cas-5B* and *QPh.cas-7D.1*, were detected (Table 4). When PH was conditioned on SL, two stable QTL *QPh.cas-2B.2* and *QPh.cas-5A.3* were detected, whereas the other ten QTL were not detected, including major QTL *QPh.cas-5A.4* and *QPh.cas-6A* (Table 4). These results indicated that SL had a significant effect on PH in PG-RIL population.

**Table 4:** Unconditional and conditional stable QTL for plant height in wheat

QTL	Env	Markers Interval	Unconditional QTL			Conditional QTL		
			PH			PH|SL		
			LOD	PVE%	Add	LOD	PVE%	Add
*QPh.cas-1A*	E1	*AX-109816727–AX-109832643*	4.009	6.730	-3.482			
	E2		3.357	3.732	-2.609	3.000	4.468	-2.440
	E3		7.572	10.230	-3.632			
	E4		8.503	8.697	-3.947			
	B		8.107	7.981	-2.945	9.676	9.599	-3.074
*QPh.cas-1B.1*	E4	*AX-110016887–AX-108733359*	2.735	2.365	1.264			
*QPh.cas-1B.2*	E3	*Xwgie50–AX-108842238*	4.336	4.214	1.718			
*QPh.cas-1B.3*	E2	*AX-108742296–AX-95142944*	5.493	6.452	1.844			
	E3					5.170	4.469	1.303
	B		7.758	6.953	1.732	5.681	6.707	1.685
*QPh.cas-2B.1*	E2	*AX-109617405–AX-108939721*	3.816	3.732	1.624			
*QPh.cas-2B.2*	E1	*AX-108853663–AX-110103130*				3.410	5.451	-2.110
	E2		4.989	5.138	-1.895	5.181	6.973	-1.877
	E3					4.692	5.622	-1.543
	B					10.07	9.520	-1.902
*QPh.cas-3A*	E4	*AX-111565008–AX-111689344*	4.022	3.533	-1.542			
*QPh.cas-3D*	B	*AX-109998069–AX-109135381*	2.873	2.484	-1.038			
*QPh.cas-4B.1*	E2	*AX-111063558–AX-109500029*	5.522	5.417	1.978	4.351	5.578	1.711
	B		4.136	3.554	1.270	3.303	2.757	1.043
*QPh.cas-4B.2*	E4	*AX-111039474–AX-108914898*	3.406	2.943	1.434			
*QPh.cas-5A.1*	E3	*AX-108846611–AX-95634783*	3.745	4.383	1.502	7.870	9.749	2.036
	B					5.869	5.487	1.446
*QPh.cas-5A.2*	E2	*AX-95628994–AX-109444105*				4.645	6.078	1.753
	E4		5.185	4.602	1.758			
	B		3.705	3.263	1.189			
*QPh.cas-5A.3*	E2	*AX-109936570–AX-110418750*	15.979	17.901	3.652	9.563	13.047	2.611
	E3		8.079	9.665	2.295	8.007	9.616	2.052
	B		9.225	8.381	1.934	10.1245	9.1657	1.897
*QPh.cas-5A.4*	E1	*AX-109514581–AX-111518796*	2.794	4.307	1.851			
	E2		3.954	3.656	1.629			
	E3		3.173	3.597	1.381			
	B		5.312	4.596	1.413			
*QPh.cas-5B*	E2	*AX-109071469–AX-109516387*				2.974	3.735	1.388
*QPh.cas-6A*	E1	*AX-108766577–AX-111257815*	6.165	9.977	2.814			
	E3		9.317	11.401	2.463			
	E4		16.826	17.037	3.439			
	B		13.679	13.248	2.397			
*QPh.cas-6B.1*	B	*AX-109863472–AX-108762584*	4.150	4.981	1.467			
*QPh.cas-6B.2*	E3	*AX-110026393–AX-109556366*	3.196	3.675	-1.392	2.692	3.069	-1.140
	B		5.833	5.298	-1.513			
*QPh.cas-6B.3*	E4	*AX-111525820–AX-108931264*	2.774	2.305	-1.265	4.214	6.472	-1.844
	B					4.012	3.558	-1.167
*QPh.cas-6B.4*	E4	*AX-111236313–AX-108763535*	4.481	3.883	1.654			
*QPh.cas-7A*	E3	*AX-108744492–AX-108738949*	3.094	3.506	-1.359	3.315	3.803	-1.270
*QPh.cas-7D.1*	E2	*AX-108882010–AX-111666703*				6.452	8.799	2.129
*QPh.cas-7D*	E1	*AX-109320176–AX-111547071*	4.369	7.262	2.402	3.866	5.992	2.218
	E3		7.714	9.459	2.238	7.293	8.900	1.946
	E4		10.259	9.721	2.600			
	B		9.064	8.421	1.912	9.294	8.570	1.809

(+) indicates that the allele is derived from the P3228, (−) indicates that the allele is derived from the G8901. E and numerals in parentheses indicate the environment in which the QTL was detected and the percentage of phenotypic variance explained (PVE) by the additive effects of the mapped QTL, respectively

When SCN was conditioned on SL, a total of 13 conditional QTL comprising 19 QTL × environments were detected for SCN|SL (Table 5). Among them, five QTL were identified by unconditional analysis, while the other 14 QTL were newly detected (Table 5). When SCN was conditioned on SL, fourteen QTL were not detected, including seven stable QTL *QScn.cas-2B.2*, *QScn.cas-4D.1*, *QScn.cas-5D*, *QScn.cas-6A.1*, *QScn.cas-6A.2*, *QScn.cas-6B*, and *QScn.cas-7A*, while the QTL *QScn.cas-3D.1*, *QScn.cas-3D.2* and *QScn.cas-6A.2* were detected (Table 5). These results suggested that SL also had a significant effect on SCN in PG-RIL population.

**Table 5:** Unconditional and conditional stable QTL for spike compactness in wheat

QTL	Marker Interval	Env	Unconditional QTL			Conditional QTL		
			SCN			SCN|SL		
			LOD	PVE (%)	Add	LOD	PVE (%)	Add
*QScn.cas-1B.1*	*AX-94442624–AX-89407680*	E3				4.735	4.082	0.227
*QScn.cas-1B.2*	*AX-108765529–AX-108864392*	E3				17.821	12.758	0.072
*QScn.cas-1B.3*	*AX-110587463–AX-109849695*	E3				22.049	16.668	-0.082
*QScn.cas-1D*	*AX-108865120–AX-109382139*	E3				5.093	2.923	0.200
*QScn.cas-2A.1*	*AX-109348712–AX-110055170*	E3	3.477	2.425	-0.044			
*QScn.cas-2A.2*	*AX-95021645–AX-111620705*	E4	3.396	2.554	-0.046			
*QScn.cas-2A.3*	*AX-111702958–AX-108747720*	E3				2.691	5.759	-0.034
*QScn.cas-2B.1*	*AX-111462859–AX-108840615*	E3		3.159	-0.050			
*QScn.cas-2B.2*	*AX-110929441–AX-110103130*	E1	8.816	11.396	-0.102			
		E3	7.695	5.893	-0.068			
		E4	8.677	6.972	-0.076			
		B	5.464	3.913	-0.050			
*QScn.cas-2D*	*AX-109316209–AX-110168677*	E4	3.452	2.519	0.046			
		B	5.745	4.413	0.054			
*QScn.cas-3A.1*	*AX-110507851–AX-109304291*	B	6.600	5.008	0.057			
*QScn.cas-3A.2*	*AX-94476859–AX-109853943*	B				3.254	5.074	-0.028
*QScn.cas-3D.1*	*AX-111064903–AX-108788717*	E1				4.876	0.906	0.055
		E2				6.659	13.387	0.052
		E3	7.414	5.474	0.066	12.758	8.412	0.058
		E4	5.864	4.505	0.061	3.492	10.151	0.041
		B	7.398	5.722	0.061	10.820	18.348	0.054
*QScn.cas-3D.2*	*AX-110834607–AX-89337262*	E1	7.255	9.809	0.094	3.393	0.649	0.047
		E3	5.611	4.377	0.059			
		E4	6.313	5.207	0.066			
		B	5.077	4.005	0.051			
*QScn.cas-4A*	*AX-109449795–AX-111102921*	B	5.924	4.510	0.054			
*QScn.cas-4D.1*	*AX-109408826–AX-108728919*	E1	4.258	5.080	0.068			
		E3	4.925	3.605	0.053			
*QScn.cas-4D.2*	*AX-111684216–AX-95658321*	E2	3.138	4.007	0.055			
*QScn.cas-5A*	*AX-108754134–AX-109324435*	B	3.253	2.418	-0.040			
*QScn.cas-5D*	*AX-111555981–AX-111262507*	E3	3.587	2.632	0.046			
		E4	7.368	5.756	0.069			
		B	6.467	5.031	0.057			
*QScn.cas-6A.1*	*AX-111504079–AX-109355289*	E3	6.208	4.656	-0.062			
		E4	4.638	3.572	-0.056			
*QScn.cas-6A.2*	*AX-108835689–AX-111257815*	E1	5.059	6.123	-0.075	62.774	27.224	-0.302
		E2	5.401	6.968	-0.072	5.409	3.245	-0.036
		B				6.056	9.916	-0.040
*QScn.cas-6B.1*	*AX-111530060–AX-111466686*	B				2.511	3.892	-0.025
*QScn.cas-6B*	*AX-111236313–AX-108763535*	E1	8.538	11.427	-0.101			
		E2	13.728	20.019	-0.123			
		E3	23.616	21.943	-0.133			
		E4	21.720	20.884	-0.134			
		B	24.495	24.213	-0.126			
*QScn.cas-6D.1*	*AX-110596748–AX-109412721*	E1	5.763	7.164	0.081			
		B	4.049	3.068	0.045			
*QScn.cas-6D.2*	*AX-111694627–AX-109997558*	E2	6.181	8.227	0.081			
		E3	11.629	9.245	0.089			
		E4	13.530	11.531	0.103			
		B	7.801	6.200	0.066			
*QScn.cas-7A.1*	*AX-108761450–AX-109338226*	E3				4.340	2.623	0.033
*QScn.cas-7A*	*AX-108786753–AX-108792313*	E1	5.280	6.544	-0.077			
		E2	5.761	7.483	-0.075			
		E3	4.905	3.492	-0.053			
		B	5.021	3.859	-0.050			
*QScn.cas-7D.1*	*AX-109352158–AX-110574768*	E3				3.592	2.100	0.030
*QScn.cas-7D.2*	*AX-108803885–AX-110969403*	E3				6.446	3.996	-0.040

(+) indicates that the allele is derived from the P3228, (−) indicates that the allele is derived from the G8901. E and numerals in parentheses indicate the environment in which the QTL was detected and the percentage of phenotypic variance explained (PVE) by the additive effects of the mapped QTL, respectively

### Important QTL clusters

A total of 11 QTL clusters were identified, and all of them were related to more than one trait (Fig. 4 and Table 6). Six intervals harboring various QTL can be identified in at least three environments (Fig. 4, Tables 3 and 6). The interval *AX-110929441*–*AX-110103130* on chromosome 2B affected PH and SL, where increased PH was contributed by the G8901 alleles, and increased SL was contributed by the P3228 alleles (Fig. 4, Tables 3 and 6). The interval *AX-111236313*–*AX-108763535* on chromosome 6B affected PH, SL, and SCN, increased PH and SL were contributed by the P3228 alleles, and increased SCN was contributed by the G8901 alleles (Fig. 4, Tables 3 and 6). The interval *AX-109320176*–*AX-111547071* on chromosome 7D showed significant effects on PH across three environments and BLUP datasets and SL in one environment. In this interval, the P3228-derived alleles increased PH and SL (Table 3). PH, SL and SCN were correlated in the PG-RIL population, it was possible that those QTL clusters were influenced by one gene with pleiotropic effects.

**Table 6:** Characterization of QTL clusters for plant height, spike length and spike compactness in this study

Clusters	Chr	Genetic Intervals (cM)	Marker Interval	QTL included	Traits (additive effect, number of environments)
C1	2B	134.726–147.365	*AX-110929441–AX-110103130*	*QPh.cas-2B.2, QSl.cas-2B.2*	PH (-1), SL (5)
C2	2D	122.055–132.439	*AX-110462142–AX-110168677*	*QSl.cas-2D.2, QScn.cas-2D*	SL (-5), SCN (2)
C3	3A	92.596–93.114	*AX-111565008–AX-111689344*	*QPh.cas-3A, QSl.cas-3A.3*	PH (-1), SL (-2)
C4	3D	76.357–92.192	*AX-109998069–AX-89337262*	*QPh.cas-3D, QScn.cas-3D.2*	PH (-1), SCN (4)
C5	4A	0.421–2.028	*AX-109449795–AX-111102921*	*QSl.cas-4A.1, QScn.cas-4A*	SL (-3), SCN (1)
C6	4D	77.985–87.273	*AX-109408826–AX-110153017*	*QSl.cas-4D, QScn.cas-4D.1*	SL (-1), SCN (2)
C7	5A	83.758–90.100	*AX-109936570–AX-110080174*	*QPh.cas-5A.3, QSl.cas-5A.2*	PH (3), SL (1)
C8	6A	50.353–65.160	*AX-111504079–AX-111257815*	*QPh.cas-6A, QScn.cas-6A.1, QScn.cas-6A.2*	PH (4), SCN (-4)
C9	6B	118.069–121.387	*AX-111236313–AX-108763535*	*QPh.cas-6B.4, QSl.cas-6B.2, QScn.cas-6B*	PH (1), Sl (5), SCN (-5)
C10	7A	96.980–101.378	*AX-109936900–AX-108792313*	*QSl.cas-7A, QScn.cas-7A*	SL (1), SCN (-4)
C11	7D	118.725–119.787	*AX-109320176–AX-111547071*	*QPh.cas-7D, QSl.cas-7D.1*	PH (4), SL (1)

a trait name in underlined type indicates that stable QTL were detected for the corresponding traits. Chr, Chromosomes. (+) indicates that the allele is derived from the P3228, (−) indicates that the allele is derived from the G8901

### Analysis of *KASP8750* alleles

**Fig. 5:**
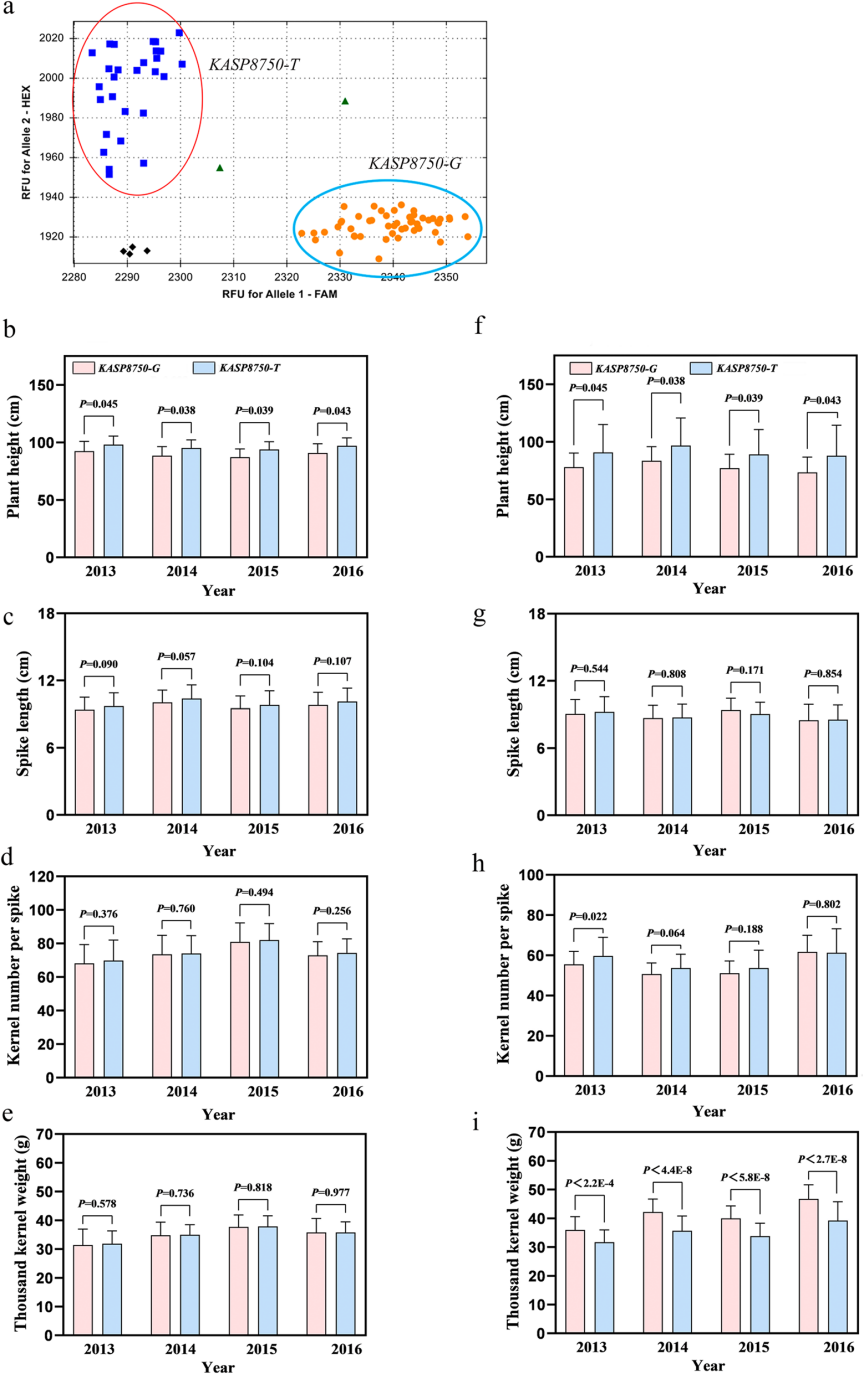
Allelic analysis with agronomic traits of *KASP8750* in PG-RIL and the natural population. **a** The allelic segregation of KASP marker *KASP8750*. Comparison analysis of *KASP8750* alleles with the plant height **b**, spike length **c**, kernel number per spike **d** and thousand kernel weight **e** of PG-RIL in four environments. Comparison analysis of *KASP8750* alleles with the plant height **f**, spike length **g**, kernel number per spike **h** and thousand kernel weight **i** of the natural population consisting of 141 cultivar/lines in four environments. ^**^P < 0.01 and ^*^P < 0.05 (two-tailed *t* test) indicates a significant difference to the two allelic

The KASP marker *KASP8750* was developed based on the SNP locus *AX-110418750* closely linked to the stable major QTL *QPh.cas-5A.3*. Two allelic effects of *QPh.cas-5A.3* were significant for the PG-RIL population and a natural population consisting of 141 cultivars/lines (Fig. 5a). After screening the PG-RIL population and the natural population using *KASP8750*, a two-tailed T test was performed between *KASP8750* and PH, SL, KNS and TKW values collected from four environments. The results showed that *KASP8750* was significantly correlated with PH but not SL, KNS or TKW for PG-RIL (Fig. 5b-e). For the natural population consisting of 141 cultivars/lines, *KASP8750* was associated with PH and TKW but not SL and KNS (Fig. 5f-i).

## Discussion

Increasing yield has been a challenging task for the breeders due to complex inheritance and quantitative nature of this trait [38]. Breeders prefer to increase the spike number per unit area by reducing PH, and increase the KNS and TKW by changing spike morphological traits such as SL and SCN, therefore, analyzing PH, SL, and SCN characters can provide specific information about genetic control and relationship between yield and its components. High diversity between parents of a population is the key point to study the genetics of a character [39]. In the current study, we used the PG-RIL population derived from the cross of P3228 and G8901, notably, those three traits were significantly different between the parents in four environments (Table 1). Transgressive segregation towards higher and lower ends of the frequency distribution for PH, SL, and SCN indicated the two parents contained different genes for the investigated traits (Table 1). The continuous distributions of the PH, SL, and SCN among PG-RIL lines and the presence of G × E interaction are certainly due to a quantitative inheritance of traits that is influenced by environment (Additional file 1: Table S1). Some studies have revealed that PH, SL and SCN are significantly affected by the environment [40, 41,32]. However, those three traits had high broad-sense heritability in PG-RIL population (Table 1), indicating adequate levels of genetic effect for these traits in the PG-RIL population. These results suggested that it was feasible and necessary to use the PG-RIL population to identify important QTL for PH, SL, and SCN.

### Comparison with previous studies

In the current study, we identified 21 QTL for PH that, five stable QTL were mainly distributed on chromosomes 1A, 5A, 6A and 7D (Table 3). Compared with the previously identified QTL, The QTL *QPh.cas-7D* for PH and *QSl.cas-7D.1* for SL in the interval *AX-109320176–AX-111547071* on chromosome 7D overlapped with *QSpl.nau-7D* (*HL2*) in the Nanda2419 × Wangshuibai RIL population [5]. moreover, the phenotype of NIL population is validated that the effect of *HL2* can increase the SL and KNS, and decrease SCN and, that is a favored morphological trait for Fusarium head blight resistance and beneficial to wheat breeding [5]. The confidence intervals of *QPh.cas-2B.2*, *QPh.cas-4B.1* and *QPh.cas-6B.3* mapped only one environment coincided with the documented *QPH.caas-2BL.1*, *QPH.caas-4BL* and *QPH.caas-6BL* in the Doumai × Shi 4185 RIL population, respectively, reflecting highly reliable QTL identification in our study. [34]. Due to the limited information of reported QTL for PH, *QPh.cas-1A* and *QPh.cas-6A* were likely novel stable QTL for PH identified in the present study.

Six stable QTL for SL were identified and, located on chromosomes 2B, 2D, 4A, and 6B (Table 3). The stable major QTL *QSl.cas-6B.2* and *QScn.cas-6B* were located in the interval *AX-108874447*–*AX-108763535* (Table 3), overlapping with *QSL.caas-6BL.1* and *QSL.saas-6B* for SL in the four RIL populations from different backgrounds [6, 34]. Notably, *QSl.cas-6B.2* also coincided with *QTKW.caas-6BL* for TKW from the Doumai × Shi 4185 RIL population [34]. These results indicate that *QSl.cas-6B.2* is a stable major QTL unaffected by genetic background that has important breeding value in wheat. *QSl.cas-1B* and *QSl.cas-2D.1* overlapped with *QSl-AxC.ipbb-1B* and *QSl-AxC.ipbb-2D.1* from the UK Avalon × Cadenza doubled haploid (DH) reference population, respectively [11]. The QTL *QSl.cas-2D.2* in the interval *AX-110462142*–*AX-110168677* on chromosome 2D has also been reported in a previous study [33]. Notably, *QSl.cas-2B.2*, *QSl.cas-4A.1* and *QSl.cas-6D.2* were likely novel QTL for SL.

Ten stable QTL for SCN were identified on chromosomes 2B, 3D, 4D, 5D, 6B, 6D, and 7A (Table 3). The stable QTL *QScn.cas-2B.2* overlapped with *QSC.cib-CK1-2B* and *QSd.sicau-2B.2* [33]. *QScn.cas-6A.1* overlapped with *QSC.cib-CK1-6A* from the Chuanmai42 × Kechengmai1 RIL population [32]. Interestingly, *QScn.cas-6A.1* was located in the same QTL cluster as *QTkw.cas-6A.1* and *QKw.cas-6A* in the PG-RIL population, which might be the major focus for breeding selection [32]. It was also reported that the stable QTL *QScn.cas-5D* coincided with *QSC.cib-CC-5D* from the Chuanmai42 × Chuannong16 RIL population [32]. Notably, *QScn.cas-3D.1*, *QScn.cas-6A.2* and *QScn.cas-7A* were likely novel stable QTL for SCN. Based on the above results, the stable QTL detected in multi-genetic background should be important selection locus in wheat breeding. Of course, the new QTL with accurate locations detected in our study need to be further verified for their genetic effects and further used in molecular assisted breeding.

The release of the hexaploid wheat reference genome has significantly accelerated the cloning of important QTL candidate genes [42,43,44]. In the current study, the stable QTL *QScn.cas-3D.2*, were located between the interval *AX-110834607*–*AX-89337262*. The gene *TaLAX1* (*TraesCS3D02G344600*), a basic helix–loop–helix transcription factor, was located in this interval. Several studies showed that loss-of-function *Talax1* mutations confer compact spikes [35]. The stable QTL *QScn.cas-4D.1* was mapped to the 466.62–476.32 Mb interval on chromosome 4DL according to the Chinese Spring reference genome v1.0 [42]. The gene *SVP3-4D* (*TraesCS4D02G301100*) was located in 469.304–469.319 Mb on 4DL. *SVP3-4D* is an important gene regulating flowering as well as wheat spike, spikelet development, and PH [36]. The stable QTL *QScn.cas-6D.2* in the interval *AX-111694627*–*AX-109997558*, was mapped to 291.14–301.71 Mb on chromosome 6D. A gene *TaPRR1-D1* (*TraesCS6D02G207100*) was located in this interval. *TaPRR1-D1* is a circadian clock gene regulating heading date, PH and TKW [37]. Those known functional genes could facilitate future studies involving positional cloning and MAS.

### Correlation between PH and SL

SL is an important factor and is highly correlated with PH. Many QTL for PH regulate SL. For instance, the important PH genes *Rht8* and *Rht25* both regulate PH and SL [7, 19]. However, several studies showed that the inheritance of QTL for PH and SL was independent of each other [45]. Conditional and unconditional QTL analyses showed that the QTL *qPH-6B* for PH was not affected by SL [41]. In the current study, conditional QTL analysis showed that *QPh.cas-5A.4* and *QPh.cas-6A* were mainly contributed by SL, while *QPh.cas-5A.3* was independent of SL (Table 4)*.* Notably, several studies showed that many QTL for SL were independently inheritanted and were not affected by PH [33, 45]. In this study, the QTL *QSl.cas-2B.2*, *QSl.cas-2D.2*, *QSl.cas-4A.1*, *QSl.cas-6D.2* and *QSl.cas-6D.2* for SL were independent of PH (Table 4). These QTL for SL could be directly used for genetic improvement of wheat spikes.

### Effects of unconditional and conditional QTL on SCN

The SCN is a composite trait determined by spikelet number per spike and SL. Conditional QTL analysis efficiently identified new QTL for SCN and revealed relationships between SCN and SL. In the present study, we identified nine new QTL for SCN on chromosomes 1B (3), 1D (1), 2A (1), 2B (1), 6B (1) and 7D (2) using conditional QTL analysis (Table 5). Fourteen QTL for SCN were not detected when SCN was conditioned on SL, indicating that the effects of these QTL were entirely contributed by SL. The unconditional QTL analysis showed that the major QTL *QScn.cas-6B* on chromosome 6B was colocalized with the QTL *QSl.cas-6B.2* for SL (Table 3). Using conditional QTL analysis, we found that *QScn.cas-6B* was entirely contributed by SL (Table 5). In conclusion, SL is the major factor affecting SCN in the PG-RIL population.

### KASP marker tightly linked to the important QTL for molecular-assisted breeding

The closely linked markers to important QTL are prerequisite in their critical for molecular-assisted selection in wheat breeding practice, which enables breeders to select favor cultivars to meet local breeding goals [46,47]. In this study, the KASP marker *KASP8750* linked to the stable QTL *QPh.cas-5A.3* was developed and verified in PG-RIL and a natural population. Recent studies show that *Rht8* and *Rht24b* have no significant negative effect on yield while reducing PH, and these dwarf genotypes have been widely used by breeders in wheat breeding [17–19]. Notably, the *KASP8750-T* allele decreased PH but did not affect SL or KNS in either PG-RIL or a natural population (Fig. 5b-i). Therefore, the KASP marker *KASP8750* will facilitate future MAS for the genetic improvement of PH in wheat.

## Conclusion

In this study, we identified 21 stable QTL in at least two individual environments. Eleven QTL clusters were identified, and all were related to more than one trait. Unconditional and conditional QTL indicated that SL is the major factor affecting SCN in the PG-RIL population. The *KASP8750-T* allele decreased PH but did not affect SL or KNS in either PG-RIL or the natural population. The user-friendly KASP marker *KASP8750* could facilitate further validation and precise introgression of potential genomic regions identified in this study through marker-assisted breeding.

## Materials and methods

### Plant material and field trials

The ‘PuBing 3228 × Gao 8901’ mapping population was used in this study to analyse the genetics of PH, SL, and SCN. The wheat germplasm P3228 has a tall PH (mean 96.95 cm), long SL (mean 10.58 cm), and low SCN (mean 2.20), whereas G8901 is a commercial cultivar with a short PH (mean 83.66 cm), short SL (mean 8.22 cm), and high SCN (mean 2.68) (Fig. 1a-b). During four growing seasons from 2013–2014 (E1), 2014–2015 (E2), 2015–2016 (E3), and 2016–2017 (E4), parents and 176 RILs were planted at the Luancheng Agroecosystem Station, Chinese Academy of Sciences (37°15″N, 114°40′47″E). In each environment, the mapping population was planted in a completely randomized block design with three replicates. Each plot consisted of a 1.5 m row with 0.25 m spacing between rows; 30 seeds were used, and 20 plants per row were retained after the emergence of seedlings through treatment. The monthly total rainfall and monthly mean temperature during the 2013–2017 in the wheat growing seasons were shown in Additional file 2: Fig. S1. Each plot received 300 kg ha^−1^ NH_4_H_2_PO_4_, 225 kg ha^−1^ CH_4_N_2_O before sowing, and another 225 kg ha^−1^ CH_4_N_2_O was top-dressed at the jointing stage. Adequate irrigation was conducted three times during the overwinter, jointing, and anthesis stages of the wheat-growing season in accordance with local standard practices. Weeds, fungal diseases, and insect pests controlled with the application of appropriate herbicides, fungicides, and insecticides, correspondingly. 

### Phenotypic evaluation and statistical analysis

For three phenotypic traits, 10 representative plants were measured from each plot to investigate PH, SL and SCN. At maturity, PH was determined as the distance between the stem base and the top of spikes (excluding awns) of the tallest culms for each plot. SL was measured from the first rachis node to the top of the uppermost spikelet excluding the awns. SCN was calculated by dividing the number of spikelets per spike by the SL.

A combined analysis of variance, mean values, standard deviations, and covariance of variation (CVs) was performed over environments for three traits were computed with SPSS Statistics v20.0 software (SPSS, Chicago, USA). Transgressive segregants were identified using least significant difference test. For each trait, the best linear unbiased predictor mean (BLUP) was calculated using the mixed linear model with the fitting of both line and environment as random effects in the lme4 package [48]. Correlation analysis of BLUP value was computed with SPSS Statistics v20.0 software (SPSS, Chicago, USA). The normal distribution of BLUP value for seven traits was tested by the Shapiro–Wilk test (α = 0.05) with SPSS Statistics v20.0 software (SPSS, Chicago, USA). Genotypic variance, environmental variance, genotypic, and environmental interaction variance were calculated using the linear model:

$${y}_{ijk}=\mu +{b}_{k/j}+{\mathrm{g}}_{i}+{e}_{j}+{\mathrm{g}e}_{ij}+{\varepsilon }_{ik},i=1,\dots ,\mathrm{g};j=1,\dots e,k=1,\dots ,r$$

For the combined ANOVA for each trait, we assume the number of genotypes is equal to *g*, the number of environments is equal to *e*, and the number of blocks is equal to *r*. Assuming *y*_*ijk*_ is the oberservation of the *I*_*th*_ genotype in the *k*_*th*_ block in the *j*_*th*_ environment. Multiple comparison tests were conducted for genotypic means in each environment by the least significance difference (LSD). Broad-sense heritability (*H*^*2*^) was calculated using the following formula *H*^*2*^ = VG/VP; where VG and VP are the genetic variance and phenotypic variance, respectively.

### QTL analysis

A high-density bin map has been constructed in our previous study [46]. QTL analysis was conducted using individual and BLUP datasets for PH, SL and SCN by inclusive composite interval mapping of additive and dominant QTL (ICIM-ADD) in QTL IciMapping v4.1 [49]. Significant QTL were determined by the LOD score at a threshold of 2.5 [50]. MapChart 2.2 (http://www.biometris.nl/uk/Software/MapChart/) was used to construct the genetic map. The QTLs were named based on McIntosh et al. [51], where ‘cas’ represents the Chinese Academy of Sciences. To identify the physical positions for the identified QTL interval, a BLAST search (http://202.194.139.32/blast/viroblast.php) was performed to align the QTL-associated flanking SNP marker sequences with the Chinese Spring reference genome v1.0 [42].

Conditional genetic analysis was conducted based on the phenotypic values of PH conditioned on SL and SCN conditioned on SL, which were obtained by the method described by Zhu [52]. The conditional phenotypic values (y_(PH|SL)_) of PH and (y_(SCN|SL)_) of SCN in wheat were obtained by the mixed-model approach. The conditional phenotypic value can be divided into y_(SCN|SL)_ = μ_(SCN|SL)_ + G_(SCN|SL)_ + E_(SCN|SL)_ + e_(SCN|SL)_, Conditional phenotypic values y_(SCN|SL)_ suggest the value of SCN without the influences of SL; μ_(SCN|SL)_ is the conditional population mean; G_(SCN|SL)_ is the conditional general genotypic effect; E_(SCN|SL)_ is the conditional effect for the environment; and e_(SCN|SL)_ is the conditional residual error. y_(SCN|SL)_ and y_(PH|SL)_ was obtained from each environment (E1, E2, E3, E4 and BLUP dataset). Conditional QTL analysis was performed to analyse the genetic contributions of SL to SCN in QTL IciMapping v4.1.

### Conversion of SNPs to KASP markers

The KASP markers were designed based on the identified SNPs obtained from the Affymetrix wheat 660 K SNP array [53], and were subsequently verified in the parents. The PG-RIL population was screened for polymorphic KASP markers. The KASP assays were performed on a BIORAD CFX96™ real-time PCR system (Bio-Rad, Hercules, CA). The reaction system employed the KASP v4.0 2 × Mastermix (LGC Genomics, Teddington, UK) and PCR conditions were based on the protocol from LGC Genomics.

## Supplementary Information

**Additional file 1:** **Supplementary file 1.**

**Additional file 2:** **Supplementary file 2.**

## Data Availability

All the data generated or analyzed during the current study were included in the manuscript and its additional files. The raw data is available from the corresponding author on reasonable request.

## Declarations

### Guideline statement

The authors confirm that all methods were carried out in accordance with relevant guidelines and regulations.
