# Supplementary material for: Identification and validation of plant height, spike length and spike compactness loci in common wheat (Triticum aestivum L.)
Source: BMC Plant Biol. 2022 Dec 6;22:568. doi: 10.1186/s12870-022-03968-0 (PMC9724413; doi:10.1186/s12870-022-03968-0)
Supplement: Supplementary file 2 — Additional file 2: Supplementary file 2. [file 12870_2022_3968_MOESM2_ESM.docx]

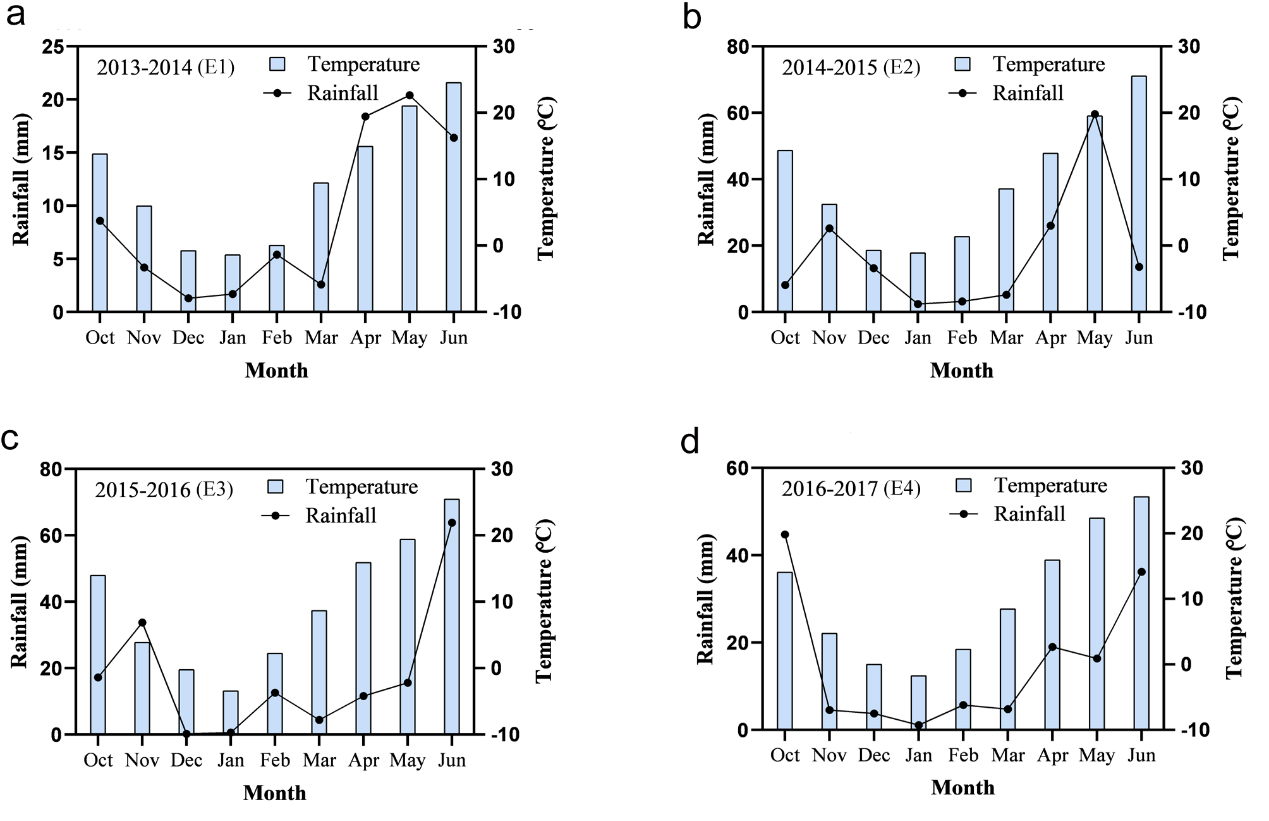


**Fig. S1** Monthly total rainfall and monthly mean temperature during the crop growing season in the experiment conducted during 2012-2013 (**a**), 2013-2014 (**b**), 2014-2015 (**c**), and 2015-2016 (**d**).
